# Impact of *Plasmodium falciparum* malaria and intermittent preventive treatment of malaria in pregnancy on the risk of malaria in infants: a systematic review

**DOI:** 10.1186/s12936-019-2943-3

**Published:** 2019-09-03

**Authors:** Abel Kakuru, Sarah G. Staedke, Grant Dorsey, Stephen Rogerson, Daniel Chandramohan

**Affiliations:** 1 Infectious Diseases Research Collaboration, P.O Box 7475, Kampala, Uganda; 2 London School of Hygiene and Tropical Medicine, Keppel Street, London, WC1E 7HT UK; 3 University of California San Francisco, San Francisco, CA USA; 4 Department of Medicine at the Doherty Institute, University of Melbourne, 792 Elizabeth Street, Melbourne, VIC 3000 Australia

**Keywords:** Malaria, Pregnancy, Infants, Intermittent preventive treatment

## Abstract

**Background:**

Studies of the association between malaria in pregnancy (MiP) and malaria during infancy have provided mixed results. A systematic review was conducted to evaluate available evidence on the impact of *Plasmodium falciparum* malaria infection during pregnancy, and intermittent preventive treatment of malaria during pregnancy (IPTp), on the risk of clinical malaria or parasitaemia during infancy.

**Methods:**

MEDLINE, EMBASE, Global Health, and Malaria in Pregnancy Library databases were searched from inception to 22 May 2018 for articles published in English that reported on associations between MiP and malaria risk in infancy. Search terms included malaria, *Plasmodium falciparum*, pregnancy, placenta, maternal, prenatal, foetal, newborn, infant, child or offspring or preschool. Randomized controlled trials and prospective cohort studies, which followed infants for at least 6 months, were included if any of the following outcomes were reported: incidence of clinical malaria, prevalence of parasitaemia, and time to first episode of parasitaemia or clinical malaria. Substantial heterogeneity between studies precluded meta-analysis. Thus, a narrative synthesis of included studies was conducted.

**Results:**

The search yielded 14 published studies, 10 prospective cohort studies and four randomized trials; all were conducted in sub-Saharan Africa. Infants born to mothers with parasitaemia during pregnancy were at higher risk of malaria in three of four studies that assessed this association. Placental malaria detected by microscopy or histology was associated with a higher risk of malaria during infancy in nine of 12 studies, but only one study adjusted for malaria transmission intensity. No statistically significant associations between the use of IPTp or different IPTp regimens and the risk of malaria during infancy were identified.

**Conclusion:**

Evidence of an association between MiP and IPTp and risk of malaria in infancy is limited and of variable quality. Most studies did not adequately adjust for malaria transmission intensity shared by mothers and their infants. Further research is needed to confirm or exclude an association between MiP and malaria in infancy. Randomized trials evaluating highly effective interventions aimed at preventing MiP, such as IPTp with dihydroartemisinin–piperaquine, may help to establish a causal association between MiP and malaria in infancy.

## Background

In sub-Saharan Africa, an estimated 30 million pregnant women are at risk of *Plasmodium falciparum* infection every year [1]. In areas of moderate to high malaria transmission intensity, like most parts of sub-Saharan Africa, *P. falciparum* infection in pregnant women is usually asymptomatic because adults are usually partially immune to malaria infection. However, *P. falciparum* infection during pregnancy can lead to placental malaria (PM). At delivery, 25% of pregnant women in sub-Saharan Africa were estimated to have PM detected by microscopy in 2007 [2]. Infection with *P. falciparum* during pregnancy has been associated with maternal morbidity such as maternal anaemia [3] and adverse birth outcomes including abortions, stillbirths, preterm delivery, and low birth weight [4–7].

The effects of *P. falciparum* infection during pregnancy on the infant may extend beyond the neonatal period [8]. Studies have shown that in utero fetal exposure to malaria antigens may negatively affect development of immunity to infectious diseases including malaria in the newborn [9–11]. Fetal exposure to *P. falciparum* antigens has been shown to induce malaria specific immune responses that are biased towards tolerance to malaria antigens [12–14] while treatment of malaria in pregnancy (MiP) was shown to be associated with pro-inflammatory responses toward malaria specific antigens [15], suggesting that infants exposed to malaria in utero may have a higher risk of malaria during early childhood and treatment of MiP may improve anti-malarial immunity in infants. However, studies evaluating the association between MiP and malaria in infancy have shown mixed results. Some studies have reported an increased risk of clinical malaria or parasitaemia in infants born to mothers with placental malaria (PM) [16–18], while others have reported no difference in the risk of malaria in infants born to mothers with and without PM at delivery [19, 20]. One study has suggested that infants born to primigravid mothers with PM have a lower risk of malaria [21].

Intermittent preventive treatment of MiP (IPTp) with sulfadoxine–pyrimethamine (SP), remains one of the main interventions recommended by the World Health Organization (WHO) in areas of moderate to high malaria transmission intensity mainly to improve birth outcomes [22] despite widespread resistance of malaria parasites to antifolate drugs [23]. Although IPTp-SP still improves birth outcomes in settings with antifolate resistance [24], its impact on PM and maternal parasitaemia remains minimal [25, 26]. This continues to expose the fetus to malaria antigens which may negatively affect the health of the infant even after delivery [27]. Intermittent preventive treatment has been shown to be associated with improved infant outcomes beyond delivery such as perinatal mortality [28] but the impact of IPTp on the risk of malaria during infancy is not well known. With available promising alternative drugs for IPTp such as dihydroartemisinin piperaquine (DP) which markedly reduce both the risk of malaria parasitaemia and incidence of clinical malaria during pregnancy and reduce the prevalence of PM at delivery but does not clearly improve birth outcomes compared to IPTp-SP [25, 26, 29], possible additional benefits of IPTp to the newborn including reducing the risk of malaria in infancy may have IPTp policy implications. Understanding the impact of IPTp on the risk of malaria in infants is important in order to maximize the benefits of malaria prevention in pregnancy.

To better understand the effect of maternal parasitaemia, PM, and IPTp on the risk of malaria in infants, which may have potential to guide policy on the choice of alternative drugs for IPTp, a systematic review was conducted to examine and summarize published studies evaluating the impact of *P. falciparum* parasitaemia in pregnancy and PM, and the effect of IPTp, on the risk of clinical malaria or parasitaemia in infants.

## Methods

This systematic review was conducted following the Preferred Reporting Items for Systematic Reviews and Meta-Analysis (PRISMA) guidelines [30]. The protocol for this systematic review was developed and registered with PROSPERO register (Registration number: CRD42018088869) prior to conducting the review.

### Selection criteria

Original research studies were included if they were published in English and evaluated associations between *P. falciparum* infection during pregnancy or IPTp and the risk of parasitaemia or incidence of malaria in infants born to HIV-uninfected pregnant women. Only randomized controlled trials (RCTs) and prospective cohort studies in which infants were followed up for at least 6 months were included. Studies involving only HIV-exposed infants, animal studies, and studies of non-falciparum malaria were excluded.

### Information sources and search strategy

MEDLINE, EMBASE, Global Health, and Malaria in Pregnancy (MiP) Library [31] databases were searched from inception to 22 May 2018. All review authors participated in developing the search strategy and AK conducted the search. MEDLINE, EMBASE and Global Health databases were searched via the Ovid interface using Medical Subject Headings (MeSH) of key search terms. The MiP library [31] was searched using key search terms, including malaria, *Plasmodium falciparum*, falciparum malaria, pregnancy, placenta, maternal, foetal, prenatal, utero, new-born, infant, child, offspring, and preschool. The search was limited to journal articles reporting human studies and published in the English language. Additional studies were identified by scrutinising reference lists of studies that met the inclusion criteria and relevant review articles. A bibliography of included studies was shared with other experts in the field of MiP to assess whether all the relevant articles had been retrieved.

### Study selection

Lists of titles and abstracts of retrieved articles were exported to Endnote and duplicates were removed. Retrieved titles and abstracts were assessed for eligibility at the level of titles and abstracts by the corresponding author. Full articles and abstracts of potentially relevant articles and those where there was uncertainty about whether to include or exclude the article were retrieved and assessed for eligibility by two independent reviewers (AK and DC). Where there was disagreement, it was resolved by discussing with the rest of the authors.

### Data extraction process

Data were extracted by one reviewer (AK) and verified by a second reviewer (DC). A data extraction matrix in an Excel spreadsheet was developed and piloted prior to data extraction. The Excel spreadsheet included the following variables: author and year of publication, country where the study was done, study period (dates of fieldwork), transmission intensity as measured by entomological inoculation rate, study design (considered RCT if IPTp was randomized, otherwise defined as an observational study), length of follow-up, follow-up schedule, study objectives, study population, sample size, long-lasting insecticide treated net (ITN) coverage among study participants, whether IPTp was given, type and frequency of IPTp regimen, whether maternal peripheral malaria parasitaemia was measured, timing of measurement of maternal peripheral malaria parasitaemia and how it was measured, PM detection, PM case definition, when and how clinical malaria or parasitaemia were detected in the infant, study outcomes, proportion of infants born to mothers with peripheral malaria parasitaemia or PM, adjustment for potential confounding factors, losses to follow-up, proportion of infants with outcomes of interest, results including effect sizes with confidence intervals and p-values, and study strengths and limitations. Disagreements between the two reviewers were resolved by discussion. Unresolved disagreements were settled by a third reviewer (either SS or GD or SR). Corresponding authors of included studies were contacted by email for any missing or unclear information. Corresponding authors who did not respond to the first email contact were contacted two more times and if they did not respond at the third contact, they were not contacted any further.

### Assessment of risk of bias

The risk of bias in individual studies was assessed by two reviewers using the Newcastle–Ottawa quality assessment scale for cohort studies [32], and the Cochrane Collaboration tool for randomized controlled trials [33]. Details of how the risk of bias was assessed in cohort studies were published in the systematic review protocol (PROSPERO Registration number: CRD42018088869). Studies were rated on the following categories: selection, comparability and outcome. For the selection category, studies were rated on the following items: representativeness of the exposed group (whether the exposed cohort was truly representative of the average from the community), selection of the non-exposed group, measurement of the exposure (if the exposed and the unexposed were from the same community), and demonstration that the outcome was not present at the start of follow-up. For the comparability category, studies were assessed based on whether the study adjusted for malaria transmission or controlled for malaria prevention during pregnancy using IPTp or ITNs. In the outcome category, studies were rated on the following items: ascertainment of outcome, whether follow-up was long enough for outcomes to occur (follow-up of at least 6 months was considered adequate), and completeness of follow-up (proportion of infants lost to follow, and whether characteristics of those lost to follow-up were reported). Each item in the selection and outcome categories was awarded a maximum of one point. The comparability category was awarded a maximum of two points. Studies were awarded a maximum of nine points. Studies that had a score of nine were rated as having medium risk of bias while those with a score of less than nine were rated as high risk.

### Data synthesis

A systematic narrative synthesis of the included studies was conducted. A meta-analysis was not conducted because studies had substantial variation in the definition of malaria exposure during pregnancy, type of IPTp given, length of follow-up, and determination of primary outcome. Results were summarized using tables and in text. Tables with summary descriptions of study design, MiP exposure measurement, follow-up time, outcome measurement and results were generated. Studies were grouped in clusters according to the type of exposure (maternal peripheral malaria during pregnancy, or PM or IPTp), and outcome of interest (incidence of malaria in infants, prevalence of malaria parasitaemia, time to first malaria parasitaemia or first episode of malaria).

## Results

### Study selection

**Fig. 1:**
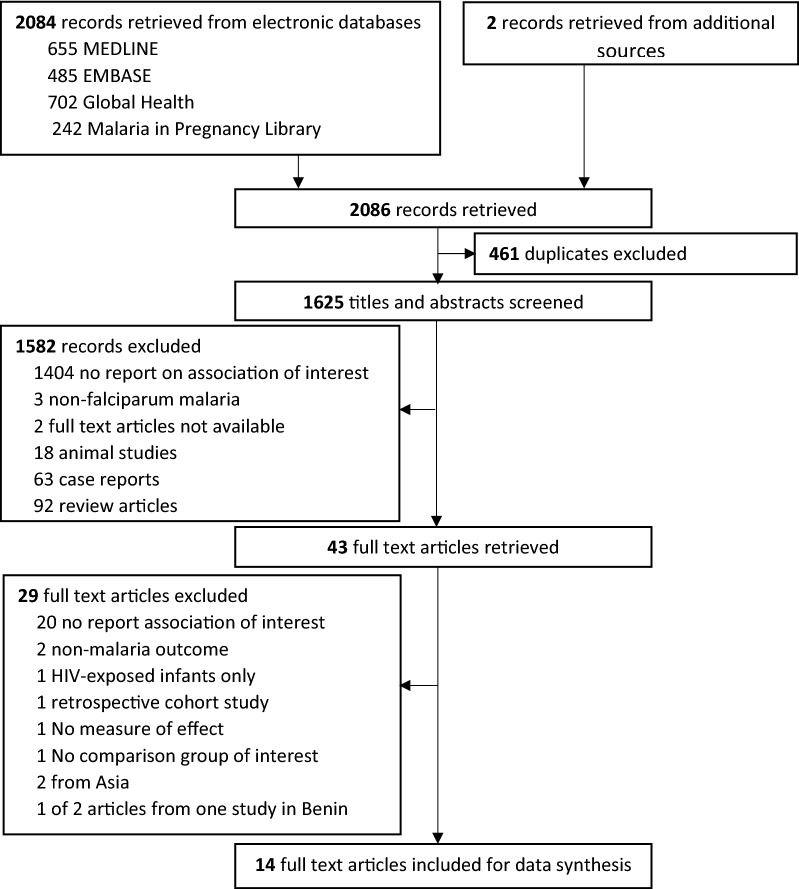
Study selection results

Overall, 2084 titles and abstracts were identified and retrieved from searches of electronic databases. An additional two records were identified from searching lists and contacting experts in the field (Fig. 1). Of the 2086 records, 461 duplicates were removed, and 1625 records were screened. Of these, 1582 records were excluded after review of the title and abstract, and 29 were excluded for various reasons after reviewing full text articles. Only 14 articles were deemed to be eligible for inclusion in the systematic review.

### Characteristics of included studies

All 14 studies were conducted in sub-Saharan Africa (Table 1); 10 prospective cohort studies and four RCTs. Of the 10 cohort studies, IPTp-SP was given in seven studies, and IPTp was not given in two [17, 34] because they were conducted before implementation of the WHO recommendation on IPTp. Malaria transmission data in form of entomological inoculation rate (number of infectious mosquito bites per person per year) was reported in half of the included studies; 20.5 [35], 35 [18], 38 [16], 50 [17], 257 [34], and 400 [19, 21]. The duration of follow-up of infants ranged from 1 to 5 years. In 10 studies, infants were followed up from birth to 1 year of age [16, 18–21, 34–38]. Maternal malaria exposure was determined by (1) microscopy (N = 5) [18, 36, 37, 39] or DNA PCR of maternal blood [40], (2) microscopy of placental blood only (N = 6) [17, 18, 21, 34, 35, 37], or (3) histology of placental tissue (N = 5) [16, 19, 36, 40, 41]. Malaria infection during infancy was measured as malaria parasitaemia (prevalence of parasitaemia, N = 7; time to first parasitaemia, N = 4), and clinical malaria (incidence of clinical malaria, N = 5; time to first clinical malaria, N = 2). Malaria parasitaemia was assessed during weekly [35], fortnightly [21], or monthly [18, 37] routine visits. Clinical malaria was assessed by active surveillance in three studies [21, 35, 36] and by passive surveillance in 11 of the studies [16–20, 34, 37–41]. ITN use in infants was not reported in half of the studies. In five studies, ITN use was reported as 100% [20, 41], 51% [21], 73% [19], and 95% [18]. In one study, 66% of infants were from families that owned at least an ITN at enrolment [35] while in another study, all infants did not have ITNs [34]. The median number of infants born to mothers with maternal parasitaemia was 224 (range 28–236). The median number of infants born to mothers with PM detected by microscopy or histology was 59 (range 15–445). Out of 11 studies which assessed association between PM and the risk of malaria in infants, there were three studies with ≥ 100 infants born to mothers with PM.

**Table 1:** Characteristics of included studies

Author, year of publication. Country (references)	N	Study design	IPTp regimens	Follow-up duration	Measures of malaria in pregnancy			Measures of malaria during infancy	
					Maternal blood^a^	Placental blood^b^	Placental histology^c^	parasitaemia	Clinical malaria
Tassi Yunga, 2018. Cameroon [34]	80	Cohort	None	1 year		✓		✓	
Boudova, 2017. Malawi [40]	473	RCT	SP vs CQ vs CQ prophylaxis	2 years	✓	✓	✓	✓	✓
Ruperez, 2016. Benin, Gabon, Tanzania, Mozambique [38]	4247	RCT	SP vs MQ	1 year					✓
Sylvester, 2016. Tanzania [41]	206	Cohort	Not reported	2 years			✓	✓	
De Beaudrap, 2016. Uganda [37]	832	Cohort	SP	1 year	✓	✓		✓	
Awine, 2016. Ghana [19]	988	RCT	SP vs ISTp-AL	1 year			✓		✓
Apinjoh, 2015. Cameroon [36]	415	Cohort	SP	1 year	✓	✓	✓	✓	
Ndibazza, 2013. Uganda [39]	2289	Cohort	SP	5 years	✓				✓
Borgella, 2013. Benin [18]	194	Cohort	SP	1 year	✓	✓		✓	✓
Asante, 2013. Ghana [20]	1855	Cohort	SP	1 year					✓
Le Port, 2011. Benin [35]	550	Cohort	SP	1 year		✓		✓	
Bardaji, 2011.Mozambique [16]	997	RCT	SP vs placebo	1 year		✓	✓	✓	
Schwarz, 2008. Gabon [17]	527	Cohort	None	2.5 years		✓			✓
Mutabingwa, 2005. Tanzania [21]	453	Cohort	SP	1 year		✓		✓	

*AL* artemether lumefantrine, *CQ* chloroquine, *IPTp* intermittent preventive treatment of malaria in pregnancy, *ISTp* intermittent screening and treatment of malaria in pregnancy, *MQ* mefloquine, *RCT* randomized controlled trial, *SP* sulfadoxine–pyrimethamine

^a^Malaria detected in maternal blood by microscopy or PCR

^b^Placental malaria detected in placental blood by microscopy

^c^Placental malaria detected in placental tissue by histology

### Association between maternal parasitaemia and the risk of malaria in infancy

The association between maternal parasitaemia and malaria risk in infancy was assessed in three cohort studies [18, 37, 39] and one RCT of IPTp-SP vs IPTp with chloroquine (CQ) vs CQ prophylaxis conducted in Malawi [40] (Table 2). In Malawi, infants born to mothers with malaria parasitaemia detected during the 2nd or 3rd trimester had higher odds of parasitaemia compared to infants born to mothers without parasitaemia during pregnancy, but the difference was not statistically significant [40]. In a cohort study conducted in Uganda, infants born to mothers with any parasitaemia detected by microscopy during pregnancy had a higher risk of parasitaemia during the first year of life compared to infants born to mothers without parasitaemia during pregnancy adjusted for gravidity, birth season, haemoglobin genotype, and residence (risk ratio [RR] 2.97; 95% confidence interval [CI] 1.37–6.42) [37]. In Benin [18], infants born to mothers having parasitaemia in the 3rd trimester had higher prevalence of parasitaemia (odds ratio [OR] 4.16; 95% CI 1.64–10.54), shorter time to first clinical malaria (hazard ratio [HR] 3.19; 95% CI 1.59–6.38) and shorter time to first parasitaemia (HR 2.95; 95% CI 1.58–5.5) compared to those born to mothers without parasitaemia in the 3rd trimester after adjusting for birth season and residence near the lake (Table 2). In the same study, maternal parasitaemia detected during 1st or 2nd trimester was not associated with an increased risk of malaria during infancy [18]. In another cohort study conducted in Uganda, infants born to mothers with malaria parasitaemia at enrolment or at delivery had a higher incidence of clinical malaria during the first 5 years of life compared to infants born to mothers without parasitaemia (HR 1.23; 95% CI 1.01–1.51) [39].

**Table 2:** Association between maternal parasitaemia and malaria risk in infancy stratified by outcome measure

Author, year of publication (refs.)	Timing of measurement of maternal parasitaemia	Method used to detect maternal parasitaemia	Exposure groups (n)	Measure of association (95% CI), *p* value	Confounders adjusted for
Prevalence of parasitaemia					
Boudova, 2017 [40]	2nd and 3rd trimester	PCR	Unexposed (n = 184)	Reference	Maternal age, gestation age at delivery, IPTp arm
			Exposed (n = 28)	OR = 1.5 (0.5–4.4), p = 0.45	
De Beaudrap, 2016 [37]	2nd and 3rd trimester	Microscopy or RDT	Unexposed (n = 626)	Reference	Gravidity, birth season, haemoglobin genotype, residence
			Exposed (n = 198)	RR = 2.97 (1.37–6.42), p = NR	
Borgella, 2013 [18]	1st trimester	Microscopy	Unexposed (n = NA)	Reference	Residence near the lake, birth season
			Exposed (n = NA)	OR = 1.12 (0.23–5.45), p = 0.89	
	2nd trimester	Microscopy	Unexposed (n = 142)	Reference	
			Exposed (n = 52)	OR = 0.87 (0.35–2.09), p = 0.75	
	3rd trimester	Microscopy	Unexposed (n = 121)	Reference	
			Exposed (n = 73)	OR = 4.16 (1.64–10.54), p = 0.003	
Time to first parasitaemia					
Borgella, 2013 [18]	1st trimester	Microscopy	Unexposed (n = NA)	Reference	Residence near the lake, birth season
			Exposed (n = NA)	HR = 1.00 (0.42–2.39), p = 0.99	
	2nd trimester	Microscopy	Unexposed (n = 142)	Reference	
			Exposed (n = 52)	HR = 1.14 (0.62–2.12), p = 0.68	
	3rd trimester	Microscopy	Unexposed (n = 121)	Reference	
			Exposed (n = 73)	HR = 2.95 (1.58–5.50), p = 0.001	
Time to first clinical malaria					
Borgella, 2013 [18]	1st trimester	Microscopy	Unexposed (n = NA)	Reference	Residence near the lake, birth season
			Exposed (n = NA)	HR = 0.97 (0.32–2.92), p = 0.95	
	2nd trimester	Microscopy	Unexposed (n = 142)	Reference	
			Exposed (n = 52)	HR = 1.15 (0.58–2.28), p = 0.70	
	3rd trimester	Microscopy	Unexposed (n = 121)	Reference	
			Exposed (n = 73)	HR = 3.19 (1.59–6.38), p = 0.001	
Incidence of clinical malaria					
Ndibazza, 2013 [39]	Baseline and delivery	Microscopy	Unexposed (n = 2053)	Ref	Maternal age, ITN possession, parity, education, social economic status, residence, mother’s HIV status
			Exposed (n = 236)	HR = 1.23 (1.01–1.51), p = 0.04	

*CI* confidence interval, *HIV* human immunodeficiency virus, *HR* hazard ratio, *IPTp* intermittent preventive treatment of malaria in pregnancy, *ITN* insecticide treated net, *NA* not applicable number was imputed, *NR* not reported, *OR* odds ratio, *RDT* rapid diagnostic test, *RR* risk ratio, *PCR* polymerase chain reaction

### Association between placental malaria and risk of malaria in infants

Six studies evaluating the association between PM detected by microscopy only and the risk of malaria in infants produced mixed results (Table 3). The prevalence of parasitaemia was higher in infants born to mothers with PM detected by microscopy compared to those born to mothers without PM (RR 10.42; 95% CI 2.64–41.10) in a cohort study conducted in Uganda [37] while the prevalence of parasitaemia and clinical malaria tended to be lower in Benin [18] though this was not statistically significant (OR 0.72; 95% CI 0.25–2.11). Compared to infants born to mothers without PM, infants born to mothers with PM detected by microscopy had a shorter time to first parasitaemia in Cameroon [34], Benin [35] and Tanzania [21], and a shorter time to first clinical malaria in Gabon (HR 2.1; 95% CI 1.2–3.7) [17]. The only study that adjusted for malaria exposure among other confounding factors showed an association between PM detected by microscopy and time to first parasitaemia but only among infants resident in houses with ITNs (HR 2.13; 95% CI 1.24–3.67) [35]. In another study conducted in Benin, there was no statistically significant association between PM detected by microscopy and time to first parasitaemia (OR 0.68; 95% CI 0.34–1.38) or time to first clinical malaria (HR 0.60; 95% CI 0.28–1.32) in infants [18].

**Table 3:** Association between placental malaria detected by microscopy and the risk of malaria in infancy stratified by outcome

Country, year of publication (ref)	PM exposure group (n)	Measure of association (95% CI), p-value	Confounders adjusted for
Prevalence of parasitaemia			
De Beaudrap, 2016 [37]	Unexposed (475)	Reference	Gravidity, maternal age, residence, level of education, season, maternal HIV status, ITN use
	Exposed (15)	RR = 10.42 (2.64–41.10), p = NR	
Borgella, 2013 [18]	Unexposed (154	Reference	Residence near the lake, birth season
	Exposed (36)	OR = 0.72 (0.25–2.11), p = 0.55	
Time to first parasitaemia			
Tassi Yunga, 2018^a^ [34]	No PM (36)	Reference	Gravidity, birth season Hb genotype, residence
	PM Lo (18)	HR = 2.6 (1.3–4.8)	
	PM Hi (18)	HR = 1.5 (0.7–3.7)	
Borgella, 2013 [18]	Unexposed (154)	Reference	Residence near the lake, birth season
	Exposed (36)	HR = 0.68 (0.34–1.38), p = 0.29	
Le Port, 2011 [35]	Unexposed (485)	Reference	Unadjusted
	Exposed (59)	HR = 1.62 (1.08–2.43), p = 0.02	
Le Port, 2011 [35]	Unexposed, had ITN (321)	Reference	Exposure to anopheles, season, antenatal care, severe anaemia
	Exposed, had ITN (34)	HR = 2.13 (1.24–3.67), p < 0.01	
	Unexposed, no ITN (158)	Reference	
	Exposed, no ITN (25)	HR = 1.18 (0.60–2.33), p = 0.62	
Mutabingwa, 2005 [21]	Unexposed (384)	Reference	Gravidity, residence, transmission season at birth, and bed net usage
	Exposed (69)	HR = 1.41 (1.01–1.99), p = NR	
Time to first clinical malaria			
Borgella, 2013 [18]	Unexposed (154)	Reference	Residence near the lake, birth season
	Exposed (36)	HR = 0.60 (0.28–1.32), p = 0.21	
Schwarz, 2008 [17]	Unexposed (477)	Reference	Gravidity, residence, birth season, IPTi, bed net use
	Exposed (50)	HR = 2.1 (1.2–3.7), p = NR	

*CI* confidence interval, *HIV* human immunodeficiency virus, *HR* hazard ratio, *IPTi* intermittent preventive treatment of malaria in infancy, *ITN* insecticide treated net, *NR* Not reported, *OR* odds ratio, *PM* placental malaria, *RR* risk ratio

^a^Placental malaria detected by microscopy or PCR; PM Lo, placental malaria with < 25 infected erythrocytes/µL; PM Hi, placental malaria with > 25 infected erythrocytes/µL

Five studies evaluated associations between PM detected by histology and the risk of malaria in infancy (Table 4). Histology detected PM was associated with an increase in the odds of clinical malaria in Malawi (OR 3.9; 95% CI 1.2–13.0) [40], Tanzania (OR 4.79; 95% 2.21–10.38) [41], and Mozambique (OR 4.63; 95% CI 2.10–10.24) [16] while one study in Cameroon did not find a statistically significant association between histologically detected PM and prevalence of malaria in infants (OR 0.72; 95% CI 0.40–1.28) [36]. Histologically detected PM was associated with a higher incidence of malaria in Malawi (unadjusted incident rate ratio [IRR] 2.3; 95% CI 1.1–4.8) [40], but this was not observed in Ghana (IRR 0.86; 95% CI 0.54–1.37) [19].

**Table 4:** Association between placental malaria detected by histology and the risk of malaria in infancy stratified by outcome

Country, year of publication (ref)	Placental malaria exposure group (n)	Measure of association (95% CI), p-value	Confounders adjusted for
Clinical malaria			
Boudova, 2017 [40]	Unexposed (184)	Reference	Maternal age, gestation age at delivery, IPTp arm
	Exposed (67)	OR = 3.9 (1.2–13.0), p = 0.03	
Boudova, 2017 [40]	Unexposed (184)	Reference	Unadjusted
	Exposed (67)	IRR = 2.3 (1.1–4.8), p = NR	
Sylvester, 2016 [41]	Unexposed (165)	Reference	Gravity, season of birth, infant birth weight, maternal age
	Exposed (41)	OR = 4.79 (2.21–10.38), p < 0.05	
Awine, 2016 [19]	Unexposed (484)	Reference	ITN use, gender, social economic status, living near an irrigated area, infant age, maternal baseline parasitaemia
	Exposed (202)	IRR = 0.86 (0.54–1.37), p = 0.52	
Apinjoh, 2015 [36]	Unexposed (n = 237)	Reference	Not indicated
	Exposed (n = 166)	OR = 0.72 (0.40–1.28), p = 0.26	
Bardaji, 2011 [16]	Unexposed (424)	Reference	Residence near the lake, birth season
	Past infection (321)	OR = 3.06 (1.94–4.82), p < 0.001	
	Acute infection (42)	OR = 4.63 (2.10–10.24), p < 0.001	
	Chronic infection (82)	OR = 3.95 (2.07–10.24), p < 0.001	
Prevalence of parasitaemia			
Boudova, 2017 [40]	Unexposed (184)	Reference	Maternal age, gestation age at delivery, IPTp arm
	Exposed (67)	OR = 2.5 (1.0–6.3), p = 0.06	

*CI* confidence interval, *IPTp* intermittent preventive treatment of malaria in pregnancy, *IRR*, incident rate ratio, *ITN* insecticide treated net, *NR* not reported, *OR* odds ratio

### Impact of IPTp on the risk of malaria in infants

Four studies evaluated the impact of IPTp on the risk of malaria in infants (Table 5). Of these, three were RCTs and one was an observational study where some women received IPTp-SP and others received no IPTp. There was no significant difference in the incidence of malaria among infants born to mothers randomized to IPTp-MQ vs IPTp-SP (IRR 0.95; 95% CI 0.81–1.13) in a multicentre RCT conducted in Benin, Gabon, Tanzania, and Mozambique [38] and among infants born to mothers randomized to intermittent screening and treatment of MiP (ISTp) with artemether–lumefantrine (AL) vs IPTp-SP (IRR 0.94; 95% CI 0.68–1.59) conducted in Ghana [19]. In a cohort study conducted in Ghana, the risk of malaria was higher in infants born to mothers who did not receive IPTp compared to infants born to mothers who received IPTp-SP, but this difference was also not statistically significant [20]. In Mozambique, the odds of clinical malaria in infants born to mothers who were randomized to IPTp-SP were higher, but not statistically significantly so, than in infants born to mothers randomized to placebo (OR 1.28; 95% CI 0.90–1.83) [16].

**Table 5:** Impact of IPTp on the risk of malaria in infancy

Author, year of publication	Randomized Y/N	IPTp arm (n)	Measure of association (95% CI), p-value	Confounders adjusted for
Ruperez,, 2016 [38]	Yes	IPTp-SP (1432)	Reference	Country
		IPTP-MQ (2815)	IRR = 0.95 (0.81–1.13), p = 0.60	
Awine, 2016 [19]	Yes	IPTp-SP (495)	Reference	Gender, social economic status, residence, irrigated area, season, ITN use, baseline parasitaemia, maternal haemoglobin
		ISTp-AL (493)	IRR = 0.94 (0.68–1.59), p = 0.76	
Asante, 2013 [20]	No	IPTp-SP (1755)	Reference	Unadjusted
		No IPTp (97)	HR = 1.23 (0.93–1.59), p = 0.15	
Bardaji, 2011 [16]	Yes	Placebo (500)	Reference	Unadjusted
		IPTp-SP (497)	OR = 1.28 (0.90–1.83), p = 0.17	

*AL* artemether lumefantrine, *CI* confidence interval, *IPTp* intermittent preventive treatment of malaria in pregnancy, *IRR* incident rate ratio, *ISTp* intermittent screening and treatment of malaria in pregnancy, *ITN* insecticide treated nets, *HR* hazard ratio, *MQ* mefloquine, *SP* sulfadoxine–pyrimethamine

### Assessment of risk of bias in individual studies

Risk of bias in individual studies was assessed using the Newcastle–Ottawa scale for observational studies (Table 6) and the Cochrane Collaboration tool for RCTs (Table 7). One study where IPTp was randomized was assessed using the Newcastle–Ottawa Scale for assessing risk of bias in observational studies because the study only assessed an association between MiP (and not IPTp regimens) and the risk of clinical malaria or parasitaemia in the infant [40]. All cohort studies had a representative exposed cohort, selected the non-exposed comparison group adequately and measured malaria exposure during pregnancy, and demonstrated that the outcome was not present at the beginning of follow-up. Most of the studies adjusted for IPTp and ITN use but only two studies [20, 35] adjusted for malaria exposure. In three of the studies, the number of participants lost to follow-up or the reasons for losses to follow-up were not reported [16, 36, 41]. Three studies had > 20% losses to follow-up [17, 36, 40]. The overall risk of bias in cohort studies was rated as high in nine studies [17, 18, 21, 34, 36, 37, 39–41] and medium in two studies [20, 35]. In all the RCTs, allocation concealment was adequate, and no trial was stopped before completion. Overall, the risk of bias in all three RCTs was low.

**Table 6:** Assessment of risk of bias for observational studies using the Newcastle–Ottawa scale

Author, year of publication (ref)	Selection				Comparability		Outcome			Total	Overall risk of bias
	REC	SNEC	ME	DON	AME	AI	AO	FL	CF		
Tassi Yunga, 2018 [34]	*	*	*	*	–	–	*	*	*	7	High
Boudova,, 2017 [40]	*	*	*	*	–	*	*	*	–	7	High
Sylvester, 2016 [41]	*	*	*	*	–	–	*	*	–	6	High
De Beaudrap, 2016 [37]	*	*	*	*	–	*	*	*	*	8	High
Apinjoh, 2015 [36]	*	*	*	*	–	–	*	*	–	6	High
Ndibazza, 2013 [39]	*	*	*	*	–	*	*	*	*	8	High
Borgella, 2013 [18]	*	*	*	*	–	*	*	*	*	8	High
Asante, 2013 [20]	*	*	*	*	*	*	*	*	*	9	Medium
Le Port, 2011 [35]	*	*	*	*	*	*	*	*	*	9	Medium
Schwarz, 2008 [17]	*	*	*	*	–	*	*	*	–	7	High
Mutabingwa, 2005 [21]	*	*	*	*	–	*	*	*	*	8	High

*REC* representativeness of the exposed cohort, *SNEC* selection of the non-exposed cohort *ME* measurement of exposure to malaria during pregnancy, *DON* demonstration that the outcome of interest was not present at the start of the study, *AME* adjusted for malaria transmission exposure *AI* adjusted for IPTp or insecticide treated net use, *AO* assessment of the outcome *FL* follow-up long enough for outcome to occur, *CF* completeness of follow-up

–, score of zero; *, score of one

**Table 7:** Assessment of risk of bias in randomized trials comparing the risk of malaria among infants who received different IPTp regimens

Criterion	Studies		
	Ruperez, 2016 [38]	Awine, 2016 [19]	Bardaji, 2011 [16]
Allocation concealment	Yes	Yes	Yes
Trial stopped early	No	No	No
Participants blinded	No	No	Yes
Study staff blinded	No	No	Yes
Infant malaria assessed blinded	Yes	Yes	Yes
Proportion of infants lost to follow-up	972/4247 (22.9%)	NR	NR
Overall risk of bias	Low	Low	Low

*NR* not reported

## Discussion

This systematic review assessed evidence evaluating the associations between MiP or IPTp and the risk of malaria infection or illness during infancy. Overall, the available evidence is of insufficient quality to confirm or rule out an association between maternal malaria infection and the risk of malaria during infancy. Most studies had small numbers of exposed infants and failed to control for possible confounding by malaria transmission intensity shared between mothers and their infants. Only one study that examined the association between malaria in pregnancy and the risk of malaria in infants controlled for malaria transmission at the level of the household [35]. In this study, time to first parasitaemia in infants born to mothers with PM was shorter in those who lived in households with ITNs, than in those living in households without ITNs. It is possible that household use of ITNs reflects underlying malaria transmission intensity, with households exposed to higher transmission more likely to use ITNs, and mothers and infants in such households at higher risk of malaria. However, secondary data analysis of the study did not find an association between PM and the risk of subsequent clinical malaria episodes [42]. This suggests that the effect of PM on the risk of malaria in infants wanes over time.

Majority of the studies included in this review showed an increased risk of clinical malaria or parasitaemia in infants born to mothers with maternal peripheral parasitaemia [37, 39], infants born to mothers with PM detected by microscopy [17, 21, 34, 35, 37] and in infants born to mothers with PM detected by histology [16, 40, 41]. These results could possibly be explained by confounding due to differences in malaria transmission intensity. Infants born to mothers with maternal peripheral parasitaemia during pregnancy or PM at delivery could be at higher risk of clinical malaria or parasitaemia because they live in an environment with higher risk of malaria transmission just like their mothers [43].

Because it would not be ethical and feasible to randomize pregnant women to exposure to MiP, alternative study designs are needed, such as randomising pregnant women to IPTp interventions with different efficacies and comparing the risks of parasitaemia and clinical malaria in the different sets of infants born to mothers who received different IPTp interventions. In this systematic review, three RCTs which evaluated the association between IPTp and the risk of malaria during infancy showed no difference in the risk of malaria among infants born to mothers who received IPTp-MQ vs IPTp-SP [38], ISTp-AL vs IPTp-SP [19], and IPTp-SP vs placebo [16]. These studies were possibly limited by the failure of the alternative intervention to significantly reduce the burden of malaria especially PM during pregnancy [44, 45]. The prevalence of PM was not significantly different among mothers randomized to IPTp-MQ (4.6%) compared to IPTp SP (5.4%, p = 0.19) in the multicentre trial [44], and was similar among mothers randomized to IPTp-SP (24.5%) compared to ISTp-AL (24.2%) in the Ghana trial [45]. In the Mozambique trial, the prevalence of any PM detected by histology or microscopy was similar among mothers on IPTp-SP (52%) or placebo (52%) [46]. Although in the same trial, the prevalence of PM detected by microscopy was higher among women on placebo (14%) compared to women on IPTp-SP (7%) [46], the risk of clinical malaria did not differ among infants born to mothers on the two IPTp arms [16]. This could possibly be due to few malaria outcomes during infancy (there were 135 first clinical malaria episodes during follow-up), which limited the power of the study.

There is currently a promising alternative drug combination for IPTp, which substantially reduces the burden of malaria during pregnancy including PM compared to SP. Although SP remains the drug recommended by the WHO for IPTp [22], its effectiveness is affected by widespread antifolate resistance [47]. In East Africa, IPTp-DP has been shown to markedly reduce the incidence of clinical malaria and the prevalence of parasitaemia during pregnancy, and the prevalence of PM at delivery compared to IPTp-SP [25, 26, 29]. One study has evaluated the impact of IPTp-DP on malaria during infancy in Uganda. In this randomized controlled trial, which examined infants receiving DP for malaria prevention, the incidence of malaria during the first 2 years of life was higher in infants born to pregnant women who received IPTp-DP (given monthly) than in those born to women who received IPTp-SP (given every 2 months). This effect was magnified in female infants [48]. The reason for this finding is unclear, but may be due to lower blood levels of piperaquine, which were observed in female infants born to mothers who received IPTp-DP [48]. Lower piperaquine levels have been associated with a higher risk of malaria in children taking DP for malaria prevention [49], but the reason why female infants born to mothers receiving IPTp-DP would have lower levels of piperaquine is unknown.

Several studies have suggested immune tolerance [9, 12, 14, 50] as one of the potential mechanisms for the observed association between MiP and the risk of malaria in infants. Evidence from laboratory studies shows that in utero exposure to malaria antigens is associated with a bias of fetal immune responses to *P. falciparum* specific [12–14] or non-malaria specific [12, 50] antigens towards anti-inflammatory responses suggesting that exposure to malaria in utero may not only affect development of malaria specific immunity in the fetus but may also affect non-malaria specific immunity. Indeed, one study has reported an increased risk of non-malaria febrile illnesses in infants born to mothers with PM compared to infants born to mothers without PM [51]. Also, PM has been associated with a reduced maternal–fetal transfer of antibodies to *P. falciparum* [52, 53], but this was not associated with an increased risk of malaria in infants [52, 53].

This systematic review had several limitations. Substantial heterogeneity in included studies was found. Studies varied in the duration of follow-up, detection of malaria exposure during pregnancy, and approaches to measuring the outcome in infants and to data analysis. Also, important raw data like the number of malaria episodes in each malaria exposure group during pregnancy was not presented in majority of the studies. Majority of studies included in this systematic review used PM as a proxy measure of MiP. PM may not be a good proxy measure of MiP because some mothers with peripheral malaria parasitaemia may clear the parasites especially with highly efficacious IPTp drugs. However, majority of the included studies used IPTp-SP, which is not highly effective at clearing parasites [54]. Only studies published in English were included in this systematic review. This could have led to missing out on potential studies published in English. However, a literature search not limiting the language to English did not yield any such studies. The search strategy was also limited to only published studies. This could have limited the number of studies with null findings, which are less likely to be published.

## Conclusion

The results of this systematic review suggest that there is insufficient evidence to confirm or exclude a causal association between MiP and the risk of malaria during infancy. Also, evidence on the impact of IPTp on the risk of malaria in infancy is inconclusive. There is need to better understand the association between MiP and malaria in infants in order to minimize the effects of MiP on malaria during infancy. Future studies of new IPTp interventions should consider not only evaluating the impact of IPTp on birth outcomes but also the potential impact of the intervention on the risk of clinical malaria or parasitaemia in infancy. This could have important policy implications on the choice of future drugs for IPTp.

## Data Availability

Not applicable.
